# A contextual ICT model to explain adoption of mobile applications in developing countries: A case study of Tunisia

**DOI:** 10.1371/journal.pone.0287219

**Published:** 2023-10-26

**Authors:** Boubaker Dhehibi, Mohamed Zied Dhraief, Aymen Frija, Hassen Ouerghemmi, Barbara Rischkowsky, Udo Ruediger

**Affiliations:** 1 Social, Economic, and Policy Research Team (SEPRT), International Center for Agricultural Research in the Dry Areas (ICARDA), Ariana, Tunisia; 2 Rural Economic Laboratory, National Agronomic Research Institute of Tunisia (INRAT), Ariana, Tunisia; 3 SEPRT, ICARDA, Ariana, Tunisia; 4 RASP, ICARDA, Addis Ababa, Ethiopia; 5 Resilient Agro-silvopastoral Systems Program (RASP), ICARDA, Ariana, Tunisia; Universidad Central de Chile, CHILE

## Abstract

In Tunisia, agriculture is the main source of livelihood for more than 75% of small and subsistence farmers with minimal use of technology. The use of information and communication technology (ICT),such as mobile applications, represents a pertinent opportunity for these smallholders to access agricultural innovation and market information and improve their farming technologies and farm management. Thus, ICT can act as a replacement to foster access to innovation for this category of farmers. Unfortunately, the underuse of mobile applications has contributed to low and slow adoption of agricultural innovation and consequently the benefits of this technology have not been attained. The purpose of this study is to identify the factors affecting the adoption of Short Message Service (SMS) through a contextual ICT model for livestock, olive crop, and beekeeping. Data were collected from 200 small-scale beekeepers, 225 olive growers, and 140 livestock breeders selected in Jendouba, Kairouan, and Zaghouan in Tunisia. The objective of this paper is to examine the factors that influence mobile applications using the partial least squares structural equation modelling technique, for livestock, olive crop, and beekeeping agricultural activities. The results showed that the final ICT-induced structural models were highly predictive of the use of SMS and its increased adoption. Factors affecting the use of SMS differed according to the farming system. The major perceived factor affecting the use of SMS was ‘observability’ for livestock farmers, ‘compatibility’ for olive growers, and ‘information quality’ for beekeepers. Understanding these factors by taking into account the specificity of the agricultural activity leads to a better understanding of the adoption of ICT tools by smallholder farmers in Tunisia.

## Introduction

In developing countries like Tunisia, the contribution of ICT to the access and use of input agricultural in information remains low. This is due to the presence of different challenges (factors) in putting the new knowledge to use [1]. These factors are related to the farmers perception, farmers’ influence on each other, information quality and the high cost of ICT services [2]. The use of mobile phone applications has helped developing countries like India, Kenya, Uganda, South Africa and Tanzania improve their agricultural productivity [3]. [4] revealed that there has been very limited study on mobile application usage by farmers. Most studies focus on mobile phone usage [5,6] and not mobile applications usage. These studies do not differentiate between using a mobile phone and the use of mobile phone applications. The most mobile applications fail because it is difficult to understand the needs of users of these apps [7]. This study aims to bridge this gap by analyzing the factors that affect the usage of agricultural mobile text messages in Tunisia.

Smallholder farmers in most developing countries face continuous challenges in accessing market information, knowledge, and skills that could improve their productivity and income [8,9]. The main challenges faced by small farmers are access to agricultural innovation in the use of modern technology and practices, access to market, access to financial services, and poor extension service delivery [4]. The extension of agricultural technology is an important means of accelerating the transformation of agricultural scientific and technological achievements and promoting agricultural modernization [10]. In facing these challenges, information and communications technology (ICT) can act as a replacement to foster access to innovation for this category of farmers. Over the last decade, ICT has become a solution to many problems of extension services. These technologies have the potential to help improve agricultural technology adoption. Information sharing through ICT can inform farmers about new technologies and market conditions, such as prices, to help them decide when and where to sell their harvests [11]. One way to effectively manage and address issues that hamper agricultural productivity and development is by farmers using ICT, such as mobile phone applications [12,13]. Studies by [14,15] showed that in areas where ICT is well utilized in agriculture, farmers can access agricultural information such as weather, recommended agronomic practices, and price information.

Several studies have hinted at positive impacts of SMSs applied to extension services. One of the examples of using ICT for the agricultural development was done by Mercy Corps Indonesia, an NGO who introduced the use of ICT for farmers’ empowerment. This institution, through Agri-Fin Mobile, provided technology and financial information services for the farmers of paddies, corns, chilies, and potatoes in various areas. This service is known as Rural Information Services that uses cellular telephone equipment in the form of Short Message Services [16,17] found that in Kenya, sending SMS messages with agricultural advice to smallholder sugarcane farmers increased yields by 11.5% relative to a control group with no messages. [18] report positive results from six randomized controlled trials (RCTs) in Kenya and Rwanda that used SMS messages to increase the use of agricultural lime to reduce soil acidity and increase yields.

In Tunisia, the population is expected to surpass 13.5 million by 2050, and agricultural production will need to increase significantly to meet this additional food demand [19]. Unfortunately, more than 75% of farmers are small, cultivate or own farmland of less than 10hectares, and these produce about 70% of all agricultural output [20]. Tunisia remains dependent on the import of several products to cover its domestic food demand, notably cereals. The trade balance did not exceed an average of 66% in the last decade [21], showing that there is a need to intensify farm productivity through the adoption of agricultural innovative technologies by small-scale farmers.

In agricultural-dependent economies, local governmental extension programmes have been the main conduit for disseminating agricultural information to farmers. These programmes have the objective of developing the technical and managerial skills of farmers and technology adoption by supporting rural adult learning and assisting farmers inbuilding their knowledge and capacities. Extension services are recognised as a critical component for technology transfer in the agricultural sector. It is expected that extension programmes will help increase farm productivity and revenue, reduce poverty, and minimize food insecurity [22]. However, agricultural extension services in Tunisia face several challenges that limit their effectiveness. The number of farms increased by 58.3% during1962–2006 while the number of agricultural extension staff decreased by 63.7% during 1990–2021 [23]. Today, there is one agricultural extension officer for every 1221 farmers compared to one for every296 farmers in 1993 [23]. The lack of human, financial, and logistical resources make it harder and more costly to visit remote areas.

In the last two decades, mobile phone coverage has spread rapidly in Africa, Asia, and Latin America. Over 60% of the population of sub-Saharan Africa, Asia, and Latin America had access to mobile phone coverage in 2009 [24]. Coinciding with this increase in mobile phone coverage has been an increase in mobile phone adoption: as of 2008, there were approximately 4 billion mobile phone subscribers worldwide, with 374 million subscriptions in Africa [24]. Mobile phones significantly reduce communication and information costs for the rural poor. This does not only provide new opportunities for rural farmers to access information on agricultural technologies, but also to use ICT in agricultural extension services [25]. The use of mobile phone applications has helped developing countries like India, Kenya, Uganda, South Africa, and Tanzania improve their agricultural productivity [26].

In Tunisia, the mobile phone industry is playing an increasingly important role in driving economic growth and digital inclusion across the country. The number of mobile subscribers grew from 4.7 million in 2008 to 8.8 million in 2018 [27]. The use of mobile applications (text message and short phone number) in Tunisian agricultural extension services was introduced by the Food Security Project in 2012–2013. Benefiting from this project, the National Institute of Field Crops provided input agricultural information via SMS to small cereal farmers (regarding various farming tips, such as when and how to irrigate and information on pests and diseases. The service is appreciated by the farmers [28]. So far, there have been very limited studies on mobile application usage by farmers in Tunisia. [29] reported that the underuse of the Short Message Services (SMS) has led to low adoption of farm input information and technological packages and consequently the benefit of this technology has not been achieved in terms of strengthening improved agricultural extension services for smallholder farmers in Tunisia. Compared to technologies considered more modern and up-to-date, SMS is more adapted to agricultural context characterised by a large part of small farmers (almost 75% less than 10 hectares). It can reach to a large audience at a relatively lower cost, it is accessible on all types of mobile phones and the message can be read by the recipient at any time compared to other features like mobile apps and voice calls [29].

Our study aims at analysing the factors that affect the use or acceptance of SMS as part of the extension services in Tunisia. It presents relevant theorical and practical contributions. This research contributes to enrich the literature review by investigating the challenges (factors) affecting farmers’ use of ICTs to access and use agricultural input information and their relationships to inform the design and delivery of this information service to small-scale farmers. In this sense, SMS models are proposed for an increased adoption of farm input information for livestock, olive crop, and beekeeping. In addition, the findings of this study would be very useful for development and extension institution to adapt the use of SMS based agricultural input information to specific context. This could improve the use of this ICT for small holder’ farmers and better dissemination of agricultural input information.

The results will be relevant for Tunisian decision-makers to make necessary improvements to enhance the use of ICT by small-scale farmers. Findings from this research will be useful for researchers, policy makers, development practitioners, and extension agents in Tunisia and other countries in similar contexts.

## ICT and agricultural input information in developing countries

Researchers have developed models to address use of ICT by farmers in developing countries. In Benin, the model by [30] identified User friendliness (simplicity), Observability, Relative Advantage, Compatibility as drivers in the use of ICT by rice farmers. [31] concentre on the development and adoption process of ICT enabled products and services by low-income group fostering the rural development of developing country like Bangladesh, and China based on the Technology Acceptance Model. In Pakistan [32], designed an ICT service for agriculture extension. It was informed by four factors: farmers’ lack of adaptable information (relevancy), economics barriers (ICT services cost), social and motivational issues and farmers’ perception. [33] emphasised that compatibility, relative advantage and complexity are the most perceived construct in the use of ICT. [34] was used an Extended Technology Acceptance Model to assess the willingness to adopt through the analysis of farmers’ perceived usefulness, ease of use, innovativeness, social influence, Information Awareness, cost, and socio-demographic factors. In Mali [35], investigates four factors (relative advantage, compatibility, simplicity and information quality) and their effects on ICTs’ use by small-scale cereal farmers in developing countries. [2] was proposed and ICT model to propose for increased adoption of farm input information by establishing seven factors (relative advantage, compatibility, simplicity, observability, social influence, cost and information quality) and their relationships. [36] identifies 6 categories of research Gaps in the literature review related to the adoption/use of ICT on agricultural input information in developing countries, i.e. Contradictory evidence [37,38], Knowledge void [39,40], Action-knowledge Conflict [30,41], Methodological conflict [31,42], Evaluation void [43,44] and Theory application void [40,43].

### Factors affecting the use of ICTs on agricultural input information

The degree of adoption of any innovative technology depends largely on its characteristics. [23] identified five characteristics that affect the rate at which an innovation is adopted: relative advantage, compatibility, simplicity/complexity, divisibility (trialability), and observability. Several empirical investigations in agriculture have studied factors related to farmers’ perceptions concerning adoption and use of ICT [2,32]. These researchers emphasised that perception is positively related to ICT adoption/use. [2,35] elaborated a systemic literature review on the factors affecting the use of ICT-based input of farm information in developing countries. These factors were regrouped into categories based on construct (i.e., characteristic’s–See Appendix A1) definitions: “relative advantage”, “compatibility” and “simplicity” constructs constitute the category “farmers’ perception and use of ICT-based farm input information”. “Observability” and “social influence” constructs were regrouped into a category named “farmers’ influence on each other”. “Use of ICT-based farm input information”, “increased adoption of farm input information”, “cost” and “information quality” constructs each constitute one category “[2].

### Relative advantage

The relative advantage of an innovation is referred to as its perceived usefulness, that is, ‘the degree to which the user believes that using a specific tool will enhance his or her productivity’ [30,45] refer to the relative advantage of innovation as its perceived usefulness, that is "the degree to which the user’s subjective probability that using a specific system will enhance his or her productivity". They found an effect of Relative Advantage on rice farmers’ use of ICT.

### Compatibility

Another important characteristic that can affect the adoption rate of an innovation is its perceived compatibility or acceptability [45]. Compatibility is the degree to which an innovation is perceived as consistent with the existing values, past experiences, and needs of potential adopters [46]. Compatibility explains the degree to which a technology is perceived to meet the needs of potential adopters [47]. It helps individuals to give meaning to a new idea so that it is regarded as more familiar [48]. A lack of compatibility in technology with individual needs may negatively affect the individual’s use of this technology [49,50]. In the context of farming mobile applications, compatibility is examined on the basis of farming style, type of phones and the operating system on a phone used by a farmer. These three attributes have to be compatible for him to use mobile apps in his farming activities. In their studies [51], found compatibility to positively influence Perceived Usefulness of information technology. In this study, it is hypothesised that compatibility has a significant impact on the Perceived Usefulness of mobile applications.

### Simplicity

Simplicity is the degree to which an innovation is perceived as relatively easy to understand and use. Any new idea may be classified on the complexity–simplicity continuum. Some innovations are clear in their meaning to potential adopters while others are not [30]. In most of the studies, simplicity is used instead of complexity as it positively affects the use of an innovation [46].

### Triability

Trialability is the degree to which an innovation may be experimented with on a limited basis [52]. Trialability of an innovation is important in minimising risk, uncertainty, and adverse consequences of innovation [53].

### Observability

Observability, also known as communicability, demonstrability, or describe ability, has been involved in many studies related to developing countries–it is the degree to which the results of an innovation are visible to others [30,52]. It positively affected the intention to adoption of ICTs on precision farming in Iran [52,53]. In another case in Mali, farmers said that other farmers come to them every month for farming advice [30,54]. The visible results of a fellow farmer using ICT drive them in utilisation of this ICT [2]. Studies on technology products found that observability had a significant effect on adoption intention of ICT [55,56].

### Social influence

Social influence is defined as the degree to which an individual perceives that other important people use the new system [57]. Thus, the major sources of information for farmers are predominantly local (e.g. neighbours, friends, and family) [58]. In Guinea [59], found that social influence was a key determinant in the use of an ICT (mobile phones).Subsequent studies on technology adoption [60,61] have used Subjective Norm and Social Influence interchangeably to explain the impact of other people’s views and opinions on the adoption of information technology. In most farming communities, especially in developing countries, social interactions exist within the farmers and would be necessary to see the impact on their perceived usefulness of mobile applications and their intention to use mobile apps [62].

### Quality information

The characteristics of the delivered information quality of ICT-based farm input information affect farmers’ use of an ICT [2]. Sometimes, although farmers have access to agricultural input information, they do not apply it because they question its effectiveness [63]. The information should be complete, relevant, accurate, timely, and appropriate [64] and lack of access to information with these qualities exposes individuals and communities to vulnerabilities and poverty [64]. To leverage the full potential of information dissemination enabled by mobile telephony along with supporting infrastructure and capacity building amongst farmers, it is essential to ensure information quality, timeliness, and trustworthiness [1].

### Cost

The high cost of ICT service constitutes a barrier to its use for agricultural input information [30]. Although many digital innovations aimed at agricultural development to enhance the lives of rural people are developing rapidly, there is a lack of good evidence to support their impact on development. In fact, for developing countries, the cost associated with using the technology, such as mobile handsets and mobile services, contributes to excluding many poor rural farmers from upgrading their agriculture information and services [65]. High cost is one factor that can dilute the advantages of accessing information by mobile phone in Bangladesh [66].The use of ICT-based farm input information by small-scale cereal farmers can provide them with information on farm inputs, which leads to higher adoption of better-quality agricultural inputs [2].

## Conceptual framework and research hypotheses

Several theories and models in the literature [2,67] have focused on analysing mental models for adoption decisions. These include the Technology Acceptance Model, the Theory of Reasoned Action, the Diffusion of Innovation Theory (DIT), the Theory of Planned Behaviour, and the Social Cognitive Theory.

This study uses DIT as the basis for use and adoption of ICT-based phone application by small-scale farmers particularly to access and use agricultural input information. Diffusion of Innovation Theory (DIT) was developed by Rogers in 1960 [68]. According to [69], “Rogers proposed that diffusion of innovation theory was to establish a foundation for researching innovation acceptance and adoption”. [70] reviewed over 508 diffusion studies before establishing Diffusion of Innovation Theory for the adoption of innovations among individuals and organisations. Rogers went ahead to explain the importance of the process and channel through which an innovation is communicated over time among the members of a social system. In an attempt to understand factors that influence adoption of ICT tools, which include mobile phone applications, DIT seem to be the most used theory (see Table 2.1) [71–73].

The DIT describes the process of change, for example, diffusion of innovations in a community. This theory attempts to predict the behaviour of individuals and social groups in the process of adoption of innovation, considering their personal characteristics, social relations, time factors, and the characteristics of the innovation [74]. According to [2], The DIT can be considered adequate within a research context of use of ICTs by small-scale farmers to adopt farm input information for three reasons; (1) it has been a beginning point for studies on the innovative use of ICT-based farm input information, (2) it fits better with the identified constructs, i.e., Relative Advantage, Compatibility, Simplicity, Observability and Use of ICT-based farm input information, than does any other technology acceptance model, including the Unified Theory of Acceptance and Use Technology(UTAUT), the Technology Acceptance Model (TAM), the Theory of Planned Behaviour (TBP) or the Theory of Reasoned Action (TRA), (3) it has been applied in the agricultural information services’ adoption/use by farmers in developing countries more than any other model. The DIT has five constructs that determine the rate of adoption: Relative Advantage, Compatibility, Complexity, Trialability and Observability [52]. Nevertheless, it does not have the constructs Information Quality, Cost or Social Influence that were supported empirically. [2] based on the DIKDAR model to extract the constructs Information Quality and Cost [64]; on the UTAUT to extract the construct Social Influence [57]; and on the Theory of Knowledge to extract the construct power as Increased adoption of farm input information [75].

**Fig 1:**
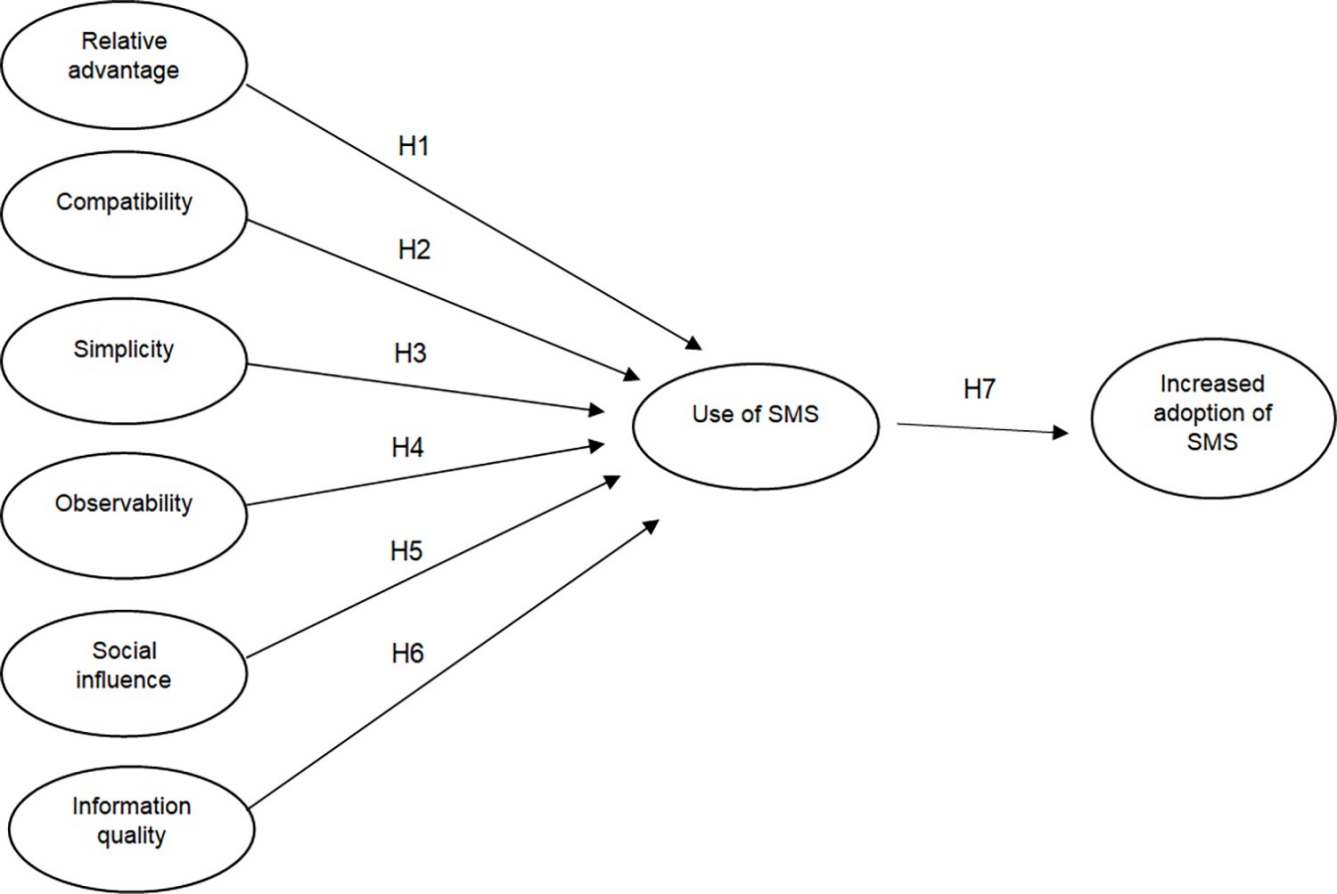
Research conceptual model, adapted from Kante et al. (2019) [2].

This study builds upon the conceptual model developed by [2] but we considered six constructs affecting the use of SMS instead of seven (Fig 1). As the SMS technology was provided to farmers for free, the ‘cost’ construct was removed from the conceptual model. The factors affecting adoption of SMS technology by farmers are summarised in the following hypotheses:

**H1.** Relative advantage has a positive impact on the use of SMS-based farm input information

**H2.** Compatibility has a positive impact on the use of SMS-based farm input information

**H3.** Simplicity has a positive impact on the use of SMS-based farm input information

**H4.** Observability has a positive impact on the use of SMS-based farm input information

**H5.** Social influence has a positive impact on the use of SMS-based farm input information

**H6.** Information quality has a positive impact on the use of SMS-based farm input information

**H7.** Use of SMS-based farm input information has a positive impact on the increase of adoption of farm input information.

## Materials and methods

### Empirical settings

This study was conducted as part of an ICARDA-led research for development project entitled ‘Project ICT2SCALE: Access to e-learning and cell-phone based services to strengthen extension services for smallholder farmers in Tunisia” implemented in Tunisia during 2019–2021.The project objective aimed to support women and men farmers to improve adoption of dryland farming technologies and farm management through the establishment of a push SMS/Unstructured Supplementary Service Data (USSD)-based system for input prices and technical information on proven technologies.

### Study area

The study focused on three governorates with similar agro-ecological conditions: Zaghouan, Jendouba, and Kairouan. Zaghouanis located in north-east Tunisia, with a total area of 2820 km^2^ and is characterised by a semiarid climate with average annual rainfall of 450 mm. In this governorate, the agricultural activities are based mainly on cereals (68400ha), olive trees (55546ha), arboriculture (5964ha), and sheep extensive farming (193000 heads female unit) as well as a recent expansion of organic crops [52]. Kairouan is in central-west Tunisia, covers an area of 6712 km^2^ and is characterised by a semiarid climate and average rainfall ranging from 200 mm in the south to 350 mm in the north. The agricultural activities are based mainly on cereals (116480ha), arboriculture (218632ha), vegetables (20858 ha), and sheep extensive farming (719000 heads female unit) [76]. The irrigated area is estimated at 58646 ha of which 25.6% belongs to the public area. The crop land use rate is about 115%. Jendoubais located innorthwest Tunisia, has an average annual temperature of 18.0°C, and 504 mm of annual precipitation. Main cereal crops grown are wheat, barley, and oats, usually integrated with small ruminants (sheep and goats). In addition to the limited natural resources, particularly arable land and water, a large number of small farmers derive most of their family income from barley/livestock-based systems, and sheep fattening practice is quite profitable in the region. Currently, the government of Jendouba and the delegations of Ain Draham, Tabarka, Fernana, and Ghardimaou within Jendouba have all the assets to develop a strong and durable beekeeping sector; more than 150 people have been recently trained in beekeeping through different projects.

### Sampling strategy and sampling procedure

The study was initiated based on a list of 700 farmers provided in 2016 by the Office of Livestock and Pasture (OEP). Farmers from Zaghouan and Kairouan (700 farmers) were chosen due to the importance of their high barley/livestock-based production systems and those from Jendouba (additional 300 farmers) due to the importance of their beekeeping activity.

Potential innovative technologies tested by the project (SMS, E-learning modules, and short phone number) were intensively discussed with National Agricultural Research and Extension Services partner organisations including the OEP and the Agricultural Training and Extension Agency (AVFA). The households were identified based on the following criteria: (i) ownership of 0–5 ha of land and (ii) ownership of 1–50 small ruminants. A total of 101 SMS messages were developed in 2019 by national experts from different agricultural domains in the following agricultural areas: cereals, forages, livestock, olives and fruit trees, vegetables, and beekeeping (honey). For each category, 10–16 messages were formulated in Arabic and French. The SMS recipients included finally1000 smallholder farmers in central and northwest Tunisia (Kairouan, Zaghouan, and Jendouba). The SMS messages were sent on a weekly basis from June 2019 for a period of 19 months.

The selection of individual farmers was based on a random sample, and a face-to-face survey was conducted in which farmers were grouped in their respective communities. The data collection process was conducted from 19 April to 8 June 2021. Farmers received relevant information (SMS) on their mobile phones. Messages were sent to all selected 1000 farmers but only 565 were surveyed. The distribution of the sample by governorates was as follows: 29% forZaghouan,42.5%forKairouan and 28.5% for Jendouba. The ICT questionnaire was divided into six modules: identification of the interviewee, SMS information, short phone number information, radio spot information, factors affecting the use of the SMS, and ICT impact on agricultural activities. For the module ‘SMS information’, a five-point scale from strongly agree (5) to strongly disagree (1) was used for assessment of the factors affecting adoption of SMS technology by farmers.

### Data analysis

Data were examined using IBM SPSS v20 involving descriptive statistics such as means and standard deviations. Data were analysed using the partial least square structural equation modelling (PLS-SEM). The PLS-SEM models are path models in which some variables may be effects of others while still affecting variables later in the hypothesised causal sequence [77]. The PLS-SEM models are an alternative to covariance-based structural equation modelling (traditional SEM) and are highly recommended in the field of information systems [35]. APLS-SEM model has two sub-model types: measurement models and the structural model. The measurement models represent the relationships between the observed data and the latent variables. The structural model represents the relationships between the latent variables. [2] summarised the criteria on the measurement of each model (Table 1).

**Table 1:** Model criteria measurements.

Measurement model assessment criteria			
Validity type	Criterion	Description	Literature
Indicator reliability	Indicator loading > 0.600	Loadings represent the absolute contribution of the indicator to the definition of its latent variable.	(Urbach and Ahlemann, 2010)
Internal consistency reliability	Cronbach’s α > 0.6	Measures the degree to which the moderating variables load simultaneously when the latent variable increases.	(Garson, 2016; Urbach and Ahlemann, 2010)
Internal consistency reliability	Composite reliability > 0.6	Attempts to measure the sum of a latent variable factor loading relative to the sum of the factor loadings plus error variance. Leads to values between 0 (completely unreliable) and 1 (perfectly reliable).	(Garson, 2016; Urbachand Ahlemann, 2010)
Content validity	Average variance extracted (AVE) > 0.5	The degree to which individual items reflecting a construct converge in comparison to items measuring different constructs.	(Garson, 2016; Henseler et al., 2016; Ahlemann, 2010)
Discriminant validity	Heterotrait-monotrait ratio (HTMT)	In information system research, it was argued that discriminant validity should be assessed by the HTMT.	(Garson, 2016; Henseler et al., 2016)
**Structural model assessment criteria**			
Model predictability	Predictive relevance Q^2^> 0.05	By systematically assuming that a certain number of cases are missing from the sample, the model parameters are estimated and used to predict the omitted values. Q^2^ measures the extent to which this prediction is successful.	(Garson, 2016; Henseler et al., 2016; Urbach and Ahlemann, 2010)
Model validity	Model fit SRMR < 1	SRMR is a measure of approximate fit of the researcher’s model.	(Garson, 2016; Henseler et al., 2016)
Model validity	R^2^> 0.100	Coefficient of determination.	(Urbach and Ahlemann, 2010)
Model validity	Path coefficients: critical t-values for a two-tailed test are 1.65 (significance level 10%), 1.96 (significance level5%), and 2.58(significance level1%)	Structural path coefficients are the path weights connecting the factors to each other.	(Garson, 2016)

Source: Adapted from Kante et al. (2019) [2].

## Results and discussion

### Sample characteristics

This section provides an overview of the socioeconomic characteristics of the 565 farmers who participated in the baseline survey in the three governorates: Zaghouan, Kairouan and Jendouba.

The results showed that 91.21% of the interviewed household heads women. Jendouba is the governorate with the highest number of women-headed households in the sample, representing 18.33% of the sample. Gender of the farmer has become an important socioeconomic variable in understanding the adoption of agricultural innovations. [78] recorded a gender gap in adoption of agricultural technologies. Previous studies showed that lack of access of women to ICT led to reduced adoption of improved agricultural technologies [79–82].Results also showed that the sample had a high level of education:25% of the sample had a university level education and only 8% were uneducated. Education of farmers is assumed to have a positive influence on farmers’ decisions to adopt innovations as it increases their ability to obtain, process, and use information relevant to adoption of a new technology [82–85].

Regarding age, the sample was divided into six classes with 14.48% aged 26–35, 22.57% aged36–45 years, 24.47% aged46–55 years,21.62% aged56–65 years and 14.48% aged more than 65 years. Youngest farmers, aged less than 26 years, represented only2.38% of the sample. Age is considered as a determinant of adoption of innovations. According to [86], younger farmers are typically less risk-averse and are more willing to try new technologies than older farmers, who have higher risk aversion and a decreased interest in long-term investment in the farm. In regard to access to phone technology, 44.18% of farmer households had a Smartphone. Almost 70% of them read SMSs regularly and 75% keep SMSs as reference information. Nevertheless, 71% of respondents declared a lack of network connectivity as the major problem in access to SMS technology.

### SMS perception of the olive growers, breeders and beekeepers

The perception of SMS was different between olive growers, breeders and beekeepers due especially to their socioeconomic and demographic characteristics. The beekeepers were the most interested by the use of SMS than the olive growers or the breeders (Appendix A2). In this sense, 31.67% of beekeepers found this technology relevant and very relevant against almost 15% for breeders and olives growers. In addition, nearly 81% of beekeepers found the information delivered by SMS of high and very high importance against 9% for olive growers and 4.22% for breeders. Concerning the usefulness of the SMS, almost all the beekeepers (96.67%) declared that the messages were useful and very useful against 16.44% for olive growers and 14.77% for breeders. Indeed, the text messages gave a lot of new information for 31.7% of beekeepers against 12.68% for breeders and 10.22% for olive growers. Nevertheless, most of beekeepers (75.83%), breeders (75.35%) and olive growers (64.44%) kept the SMS as reference information.

The preference of the beekeepers for the use of SMS can be explained by their socioeconomic and demographic characteristics (Appendix A2). Compared to the breeders and olive growers, the beekeepers were composed by more women (18.33% female against 4.89% and 3.52% respectively),the youngest ones (69.17% less than 45 years against almost 26% for breeders and olive growers), the most educated (46.67% of beekeepers having a university education against 16% and 9.86% respectively) and the most involved in the cooperatives (37.5% against 6.34% and 6.66% respectively). In addition, the beekeepers had the highest percentage of Smart phone owners (60.83% against 52.22% for olive growers and 49.10% for breeders) and the high frequencies of the SMS reception (33.33% once or twice a week against 22.22% for olive growers and 16.9% for breeders.

Compared to the beekeepers that did not have problems receiving SMS, most of the olive growers (77.27%) and the breeders (71.43%) stated the network problem as the main type of problem leading to not receive the SMS (Appendix A2). Furthermore, almost 93% the beekeepers declared reading the messages regularly against 56% for olive growers and 59.15% for breeders who mentioned a lack of motivation as the main reason for rarely or never read the SMS.

To ensure the longevity of the use of SMS, less than half of beekeepers (46.7%) agreed to pay 0.03 TND per message once the project ends against 33.78% for olive growers and 38.00% for breeders (Appendix A2). The reasons for unwillingness to pay were mostly related the problem of SMS content (information quality) and the SMS cost (SMS expensive, Extension services are free).

### Statistical analysis of variables

The statistical analyses of variables for livestock, olives, and beekeeping are presented in Tables 2–4, respectively. The objective of this analysis was to test the normality distribution of the three databases to justify the use of PLS-SEM in this study. The skew and kurtosis values showed that the three data distributions were not within the acceptable limitsof±1 [87], indicating a non-normal distribution. Nevertheless, [77] states that it is possible to use PLS path modelling with highly skewed data and argues that all SEM techniques are quite robust against a skewness scenario.

**Table 2:** Statistical analysis of variables for the livestock model.

Construct	Items	Mean	Std. error	Kurtosis	Skewness
Compatibility	COM1	1.914	1.322	−0.463	1.029
	COM2	2.439	1.546	−1.464	0.393
	COM3	1.719	1.018	−0.632	0.960
Increase adoption	INCADOP1	1.576	1.011	0.828	1.525
	INCADOP2	1.576	1.018	0.749	1.511
	INCADOP3	1.676	1.120	0.268	1.351
	INCADOP4	1.604	1.070	0.956	1.558
Information quality	IQ1	1.576	1.144	1.630	1.764
	IQ2	1.820	1.277	0.100	1.245
	IQ3	1.806	1.205	0.187	1.229
Observability	OBS2	1.885	1.342	−0.829	0.990
	OBS3	2.022	1.360	−1.141	0.759
Relative advantage	RA1	2.072	1.492	−0.772	0.927
	RA2	1.827	1.156	0.286	1.218
Social influence	SI1	2.058	1.296	−1.156	0.673
	SI2	2.259	1.624	−1.339	0.663
	SI3	1.252	0.576	5.617	2.426
Use of SMS	USMS1	2.266	1.643	−1.266	0.707
	USMS2	2.583	1.775	−1.635	0.417
	USMS3	2.446	1.623	−1.492	0.455

Source: Own elaboration from survey data (2022).

**Table 3:** Statistical analysis of variables for the olive crop model.

Construct	Items	Mean	Std. error	Kurtosis	Skewness
Compatibility	COMP1	1.839	1.296	−0.206	1.157
	COMP2	2.438	1.571	−1.367	0.467
	COMP3	1.621	0.951	−0.102	1.169
Increase adoption	INCADOP1	1.522	0.982	1.313	1.676
	INCADOP2	1.500	0.973	1.587	1.757
	INCADOP3	1.634	1.102	0.777	1.490
	INCADOP4	1.576	1.071	1.342	1.658
Information quality	IQ1	1.522	1.081	2.094	1.873
	IQ2	1.754	1.183	0.022	1.219
	IQ3	1.759	1.174	0.593	1.361
Relative advantage	RA1	1.933	1.405	−0.246	1.141
	RA2	1.799	1.169	0.336	1.259
Use of SMS	USMS1	2.201	1.598	−1.090	0.785
	USMS2	2.500	1.711	−1.494	0.490
	USMS3	2.379	1.577	−1.329	0.540

Source: Own elaboration from survey data (2022).

**Table 4:** Statistical analysis of variables for the beekeeping model.

Construct		Mean	Std. error	Kurtosis	Skewness
Observability	OBS1	3.108	1.537	−1.411	−0.073
	OBS2	1.983	1.183	−0.207	0.858
	OBS3	3.150	1.547	−1.423	−0.090
Increase adoption	INCADOP3	2.317	1.544	−1.042	0.708
	INCADOP4	1.817	1.008	−0.297	0.822
Information quality	IQ1	2.658	1.696	−1.612	0.342
	IQ2	3.142	1.561	−1.443	−0.119
	IQ3	3.158	1.511	−1.402	−0.097
Use of SMS	USMS1	3.475	1.360	−0.835	−0.410
	USMS2	3.942	1.192	−0.392	−0.752
	USMS3	3.675	1.219	−0.808	−0.359

Source: Own elaboration from survey data (2022).

### Measurement model validation

In this section, the construct validity of the three models was validated through convergent validity and discriminant validity.

#### Convergent validity

The assessment of the convergent validity was conducted on the measures of some indicators: composite reliability, Cronbach’s alpha, average variance extracted (AVE), and indicator reliability.

*Composite reliability*. The composite reliability of each construct was greater than 0.830 for livestock (Table 5), greater than 0.900 for olive crop (Table 6), and greater than 0.890 for beekeeping (Table 7).

**Table 5:** Convergent validity for the livestock model.

Construct	Items	Indicator reliability	Cronbach’s alpha	Composite reliability	AVE
**Compatibility**	COM1	0.812	0.862	0.916	0.784
	COM2	0.803			
	COM3	0.736			
**Increase adoption**	INCADOP1	0.691	0.949	0.963	0.868
	INCADOP2	0.665			
	INCADOP3	0.745			
	INCADOP4	0.774			
**Information quality**	IQ1	0.677	0.892	0.933	0.823
	IQ2	0.764			
	IQ3	0.785			
**Observability**	OBS2	0.862	0.948	0.975	0.950
	OBS3	0.861			
**Relative advantage**	RA1	0.853	0.817	0.915	0.844
	RA2	0.677			
**Social influence**	SI1	0.847	0.709	0.832	0.624
	SI2	0.538			
	SI3	0.523			
**Use of SMS**	USMS1	0.956	0.962	0.975	0.929
	USMS2	0.974			
	USMS3	0.961			

Source: Own elaboration from livestock model results (2022).

**Table 6:** Convergent validity for the olive crop model.

**Construct**	**Items**	**Indicator reliability**	**Cronbach’s alpha**	**Composite reliability**	**AVE**
Compatibility	COM1	0.821	0.843	0.905	0.761
	COM2	0.792			
	COM3	0.715			
Increase adoption	INCADOP1	0.682	0.937	0.955	0.841
	INCADOP2	0.641			
	INCADOP3	0.731			
	INCADOP4	0.752			
Information quality	IQ1	0.668	0.897	0.936	0.829
	IQ2	0.770			
	IQ3	0.814			
Relative advantage	RA1	0.830	0.838	0.924	0.859
	RA2	0.674			
Use of SMS	USMS1	0.955	0.959	0.973	0.924
	USMS2	0.969			
	USMS3	0.959			

Source: Own elaboration from olive crop model results (2022).

**Table 7:** Convergent validity for the beekeeping model.

**Construct**	**Items**	**Indicator reliability**	**Cronbach’s alpha**	**Composite reliability**	**AVE**
Observability	OBS1	0.722	0.836	0.899	0.747
	OBS2	0.653			
	OBS3	0.766			
Increase adoption	INCADOP3	0.699	0.824	0.919	0.850
	INCADOP4	0.607			
Information quality	IQ1	0.749	0.921	0.950	0.864
	IQ2	0.790			
	IQ3	0.805			
Use of SMS	USMS1	0.922	0.855	0.912	0.776
	USMS2	0.863			
	USMS3	0.856			

Source: Own elaboration from beekeeping model results (2022).

*Cronbach’s alpha*. The Cronbach’s alpha of each construct was greater than 0.810 for livestock (Table 5), greater than 0.830 for olive crop (Table 6), and greater than 0.820 for beekeeping (Table 7).

*AVE*. The AVE of each construct was greater than 0.620 for livestock (Table 5), greater than 0.760 for olive crop (Table 6), and greater than 0.740 for beekeeping (Table 7).

*Indicator reliability*. The indicator reliability of each item was greater than 0.6 for all constructs except for SI2 (0.538) and SI3 (0.523) for the livestock model (Table 5). In this sense, [88] stated that low cut-offs such as 0.4 can be accepted in an exploratory setting.

Based on these indicators, the convergent validity of each of the constructs for the three models was established.

#### Discriminant validity

In PLS-SEM, discriminant validity was assessed using three methods: AVE of Fornell–Larcker, cross-loading, and heterotrait-monotrait ratio (HTMT) [77,89,90]. However, the use of the HTMT is highly recommended in assessing discriminant validity [91]. The HTMT criterion (which should be below 1.0) was validated for all constructs for the three models (Tables 8–10). Thus, the discriminant validity of each construct for the three models was established.

**Table 8:** HTMT criteria for the livestock model.

	COM	INCADOP	IQ	OBS	RA	SI	USMS
Compatibility (COM)	0.000	-	-	-	-	-	-
Increase adoption (INCADOP)	0.773	0.000	-	-	-	-	-
Information quality (IQ)	0.885	0.895	0.000	-	-	-	-
Observability (OBS)	0.905	0.791	0.805	0.000	-	-	-
Relative advantage (RA)	0.896	0.837	0.928	0.840	0.000	-	-
Social influence (SI)	0.940	0.817	0.969	0.802	0.866	0.000	-
Use of SMS (USMS)	0.973	0.808	0.883	0.925	0.938	0.969	0.000

Source: Own elaboration from livestock model results (2022).

**Table 9:** HTMT criteria for the olive crop model.

	COM	INCADOP	IQ	RA	USMS
Compatibility (COM)	0.000	-	-	-	-
Increase adoption (INCADOP)	0.811	0.000	-	-	-
Information quality (IQ)	0.890	0.904	0.000	-	-
Relative advantage (RA)	0.889	0.860	0.929	0.000	-
Use of SMS (USMS)	0.990	0.807	0.889	0.905	0.000

Source: Own elaboration from olive crop model results (2022).

**Table 10:** HTMT criteria for the beekeeping model.

	INCADOP	IQ	OBS	USMS
Increase adoption (INCADOP)	0.000	-	-	-
Information quality (IQ)	0.853	0.000	-	-
Observability (OBS)	0.965	0.980	0.000	-
Use of SMS (USMS)	0.840	0.942	0.976	0.000

Source: Own elaboration from beekeeping model results (2022).

### Structural model

#### The structural model represents the causal model

The structural model assesses whether the measurement model is acceptable. [77] argues that the outer (measurement) model defines the meaning of the constructs in the structural model.

The primary criterion for the evaluation of the causal model is the coefficient of determination (R^2^), the second criterion is the path coefficient (β), the third is the effect size, and the fourth is the predictive relevance (Q^2^). The last criterion tests if there are any moderating variables [2].

#### R^2^

**Fig 2:**
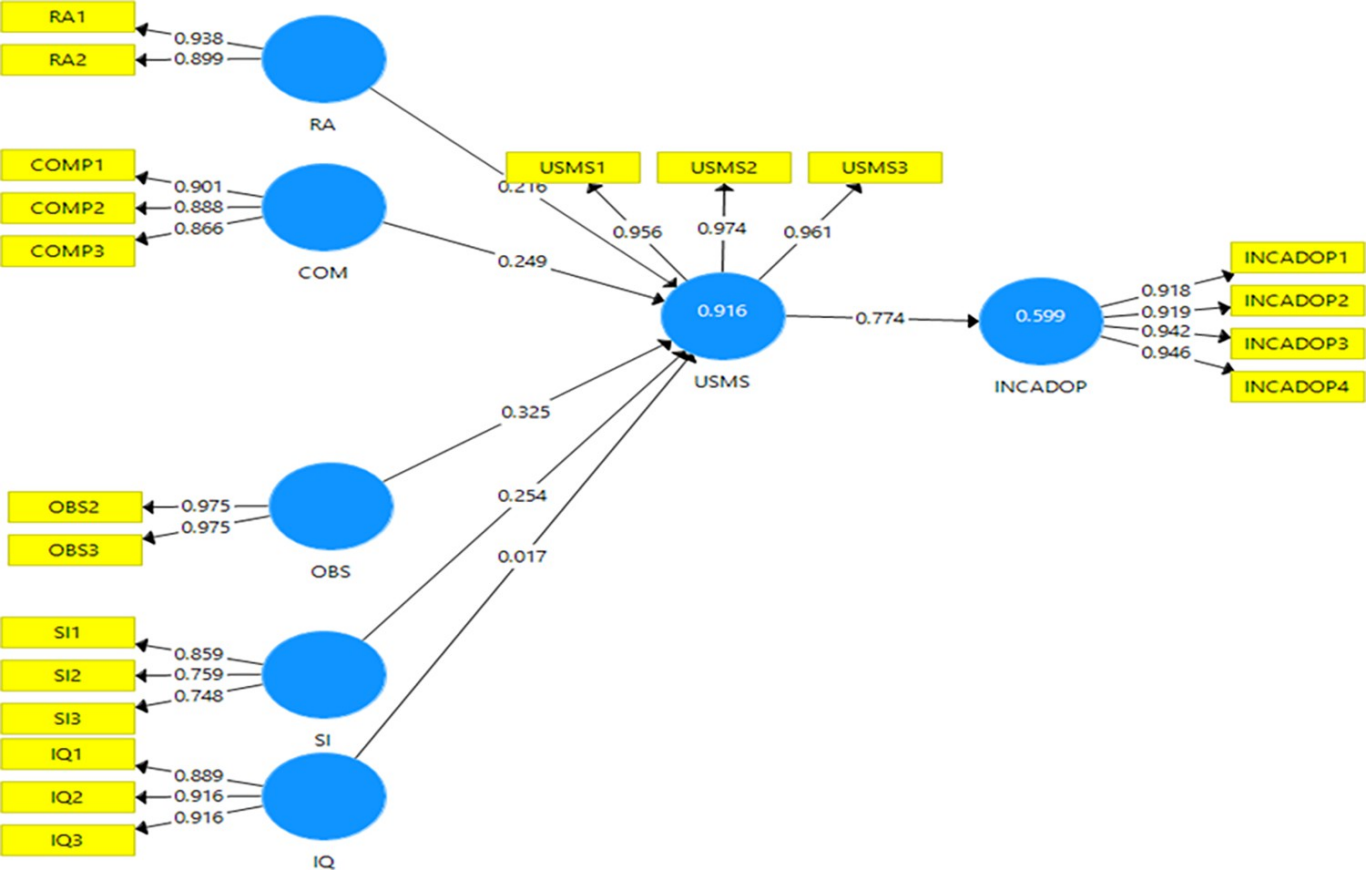
Livestock model results.

**Fig 3:**
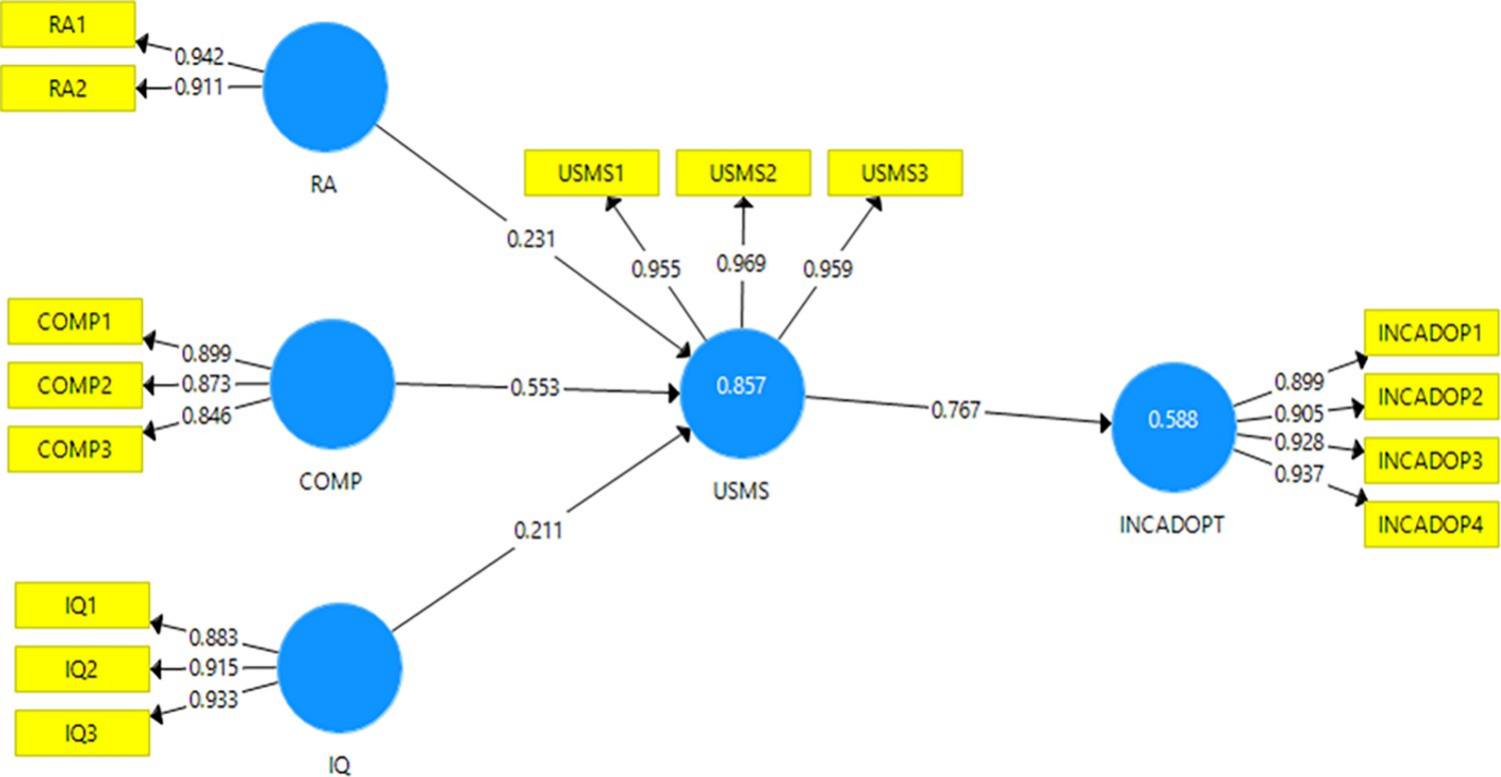
Olive crop model results.

**Fig 4:**
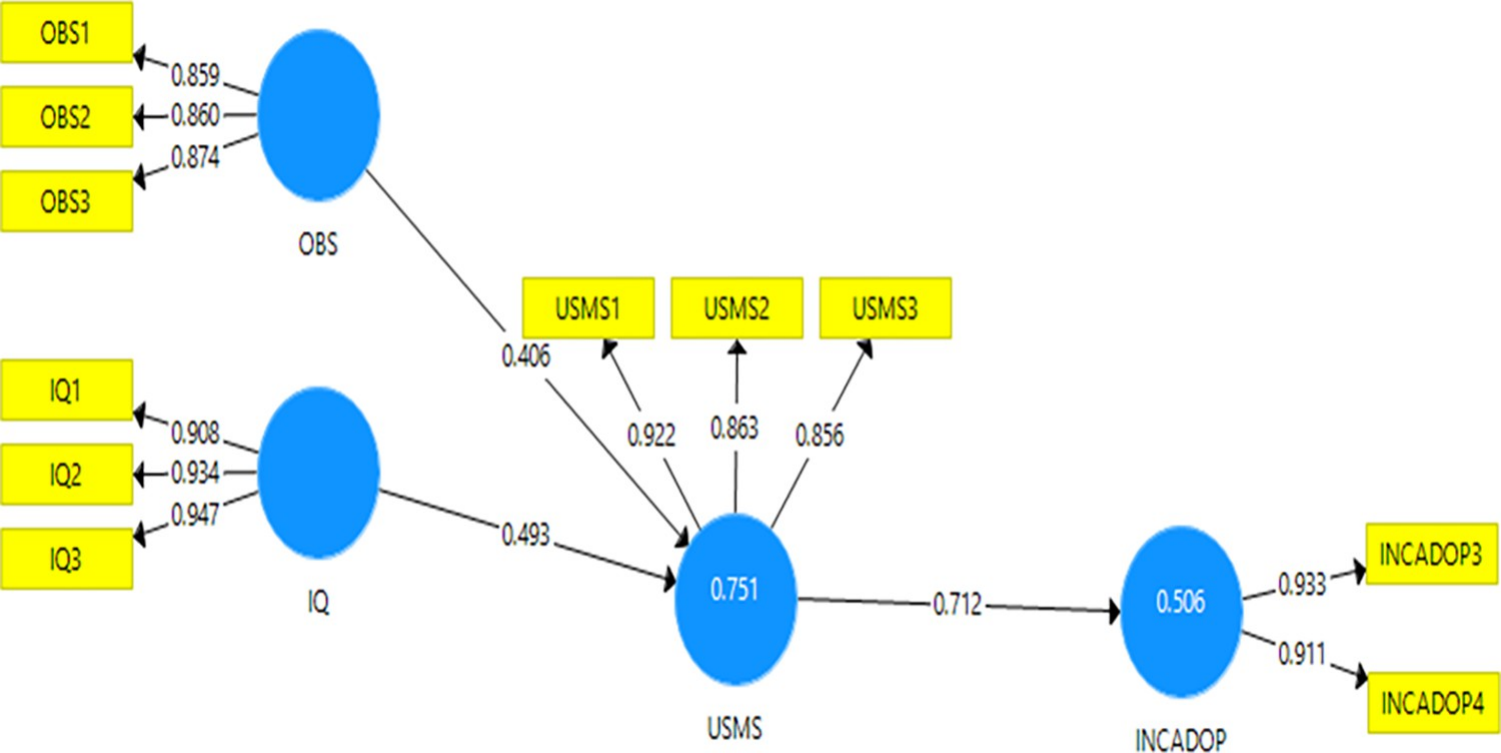
Beekeeping model results.

TheR^2^measures the proportion of the variance of the dependent variable about its mean that is explained by the independent variable(s) [92].The variance of the first endogenous variable use of SMS (i.e., USMS) was 0.916 for the livestock model, 0.857 for olive, and 0.751 for beekeeping (Figs 2–4). These R^2^ values approximated those found by [30] but were higher than for [93,94]. These findings show that the variables selected in our study for the three models explained more clearly the use of SMS technology than do the previous studies [93,94]. In addition, the latent variable use of SMS explained 0.599, 0.588, and 0.504 of the variances of increased adoption of SMS (i.e., INCADOP) for livestock, olive, and beekeeping models, respectively (Figs 2–4).

#### Path coefficient

According to [54], the weights closest to an absolute value of 1 reflect the strongest paths, while weights closest to 0 reflect the weakest paths. For the livestock model on the first endogenous variable (Table 11), observability had the strongest effect on USMS (0.325), followed by social influence (0.254), compatibility (0.249), and relative advantage (0.216) (Table 11). Information quality was not significant, with path coefficient β lower than 0.1.On the last endogenous variable; USMS had a strong effect (0.774) on INCADOP (Fig 2).

**Table 11:** Path coefficients for the livestock model.

	β	t-statistics	P-values
COM → USMS	0.249	3.570	0.000
IQ → USMS	0.017	0.268	0.789
OBS → USMS	0.325	4.844	0.000
RA → USMS	0.216	3.208	0.001
SI → USMS	0.254	4.653	0.000
USMS → INCADOP	0.774	21.861	0.000

Source: Own elaboration from livestock model results (2022).

For the olive model, three variables were significant and had an effect on the first endogenous variable (Table 12): compatibility (0.553), relative advantage (0.231), and information quality (0.211). On the last endogenous variable, USMS had a strong effect (0.767) on INCADOP (Fig 3).For the bee keeping model (Table 13), only two variables had a strong effect on USMS: information quality (0.493) and observability (0.406). On the last endogenous variable, USMS had a strong effect (0.712) on INCADOP (Fig 4).

**Table 12:** Path coefficients for the olive crop model.

	β	t-statistics	P-values
COM → USMS	0.553	9.513	0.000
IQ → USMS	0.211	3.405	0.001
RA → USMS	0.231	3.112	0.002
USMS → INCADOP	0.767	25.622	0.000

Source: Own elaboration from olive crop model results (2022).

**Table 13:** Path coefficients for the beekeeping model.

	β	t-statistics	P-values
IQ → USMS	0.493	4.453	0.000
OBS→ USMS	0.406	3.695	0.000
USMS → INCADOP	0.712	18.059	0.000

Source: Own elaboration from beekeeping model results (2022).

#### Predictive relevance

The observability, social influence, compatibility, and relative advantage, for the livestock model, were highly predictive of USMS on agricultural input information with a high Q^2^ (0.923). The USMS was also highly predictive of its INCADOP with a strong Q^2^ (0.596).For the olive crop model, the compatibility, relative advantage, and information were highly predictive of USMS on agricultural input information with a high Q^2^ (0.922). The USMS was also highly predictive of its INCADOP with a strong Q^2^ (0.586). Finally, for the beekeeping model, the information quality and observability were highly predictive of USMS on agricultural input information with a high Q^2^ (0.791). The USMS was also highly predictive of its INCADOP with a strong Q^2^ (0.550).

#### Standardised root mean square residual (SRMR)

The SRMR measures the approximate fit of the studies model. The cut-off value of SRMR of less than 1.0 is considered a good fit [54,72].Using SMARTPLS, the function model provided us with the SRMR values: 0.092 for livestock, for 0.094 olive crop, and 0.086 for beekeeping models. The studied models passed this test.

### Hypotheses validation and discussion

In the following section, the final estimated model frameworks are presented (Annex 3- Figs A3.1-A3.3 in S1 Appendix) and results of the study hypotheses based on their effect on the first and last endogenous variables by activity (Livestock, olive crops, beekeeping) will be discussed (Table 14).

**Table 14:** Hypothesis validation.

Hypotheses	Livestock model	Olive crop model	Beekeeping model
**H1.** Relative advantage has a positive impact on the use of SMS-based farm input information	**Supported**	**Supported**	**-**
**H2.** Compatibility has a positive impact on the use of SMS-based farm input information	**Supported**	**Supported**	**-**
**H3.** Simplicity has a positive impact on the use of SMS-based farm input information	**-**	**-**	**-**
**H4.** Observability has a positive impact on the use of SMS-based farm input information	**Supported**	**-**	**Supported**
**H5.** Social influence has a positive impact on the use of SMS-based farm input information	**Supported**	**-**	**-**
**H6.** Information quality has a positive impact on the use of SMS-based farm input information	**Rejected**	**Supported**	**Supported**
**H7.** Use of SMS-based farm input information has a positive impact on the increase of adoption of farm input information	**Supported**	**Supported**	**Supported**

Source: Own elaboration from livestock, olive crop, and beekeeping model results (2022).

#### Relative advantage

The first hypothesis H1 (Relative advantage has a positive impact on the use of SMS-based farm input information) was supported for the livestock and olive crop models. However, this factor was removed from the constructs for the beekeeping model. These findings confirmed for small-scale farmers growing olive crop or breeding livestock that SMS technology was better than other traditional tools. The SMSs were more interesting and contributed to the adoption of farm input information. These results were consistent with the literature for which the construct is a driver in the use of ICT-based farm input information [2,70];this factor has a significant relationship with the use of innovation [95], and relative advantage was previously found to be a driver of e-health innovation [46] and for other domains such as agriculture, education, and e-government [33]. Most of the studied small farmers stated that SMS technology significantly improved their ability to access and use farm input information compared toother available sources of information (e.g., extension services, research projects, and radio spots). However, neighbours, other farmers, and the local market remain the main sources of information for the respondents.

#### Compatibility

The second hypothesis H2 (Compatibility has a positive impact on the use of SMS-based farm input information) was supported for the livestock and olive crop models. These findings indicate the importance of new technologies being compatible with local farmer’s context (mobile phone characteristics, network availability, and connectivity). This is consistent with the findings of [47,96], who found the more compatible the technology with the existing situation of the farms, the more easily farmers will perceive it as useful, and thus it becomes more likely that they will adopt it. This construct is a driver for the use of ICT in agricultural input information and was validated as a facilitating condition by [57,95] to enhance use and adoption. This factor has a positive and significant effect on the intention to use the mobile phone [97–100]. In contrast, the compatibility construct was removed from the beekeeping model. This result can be explained by the fact that beekeepers’ responses related to the compatibility items were contradictory:41.67% totally disagreed that SMS is suitable to the way they like to get information on farm inputs, 67.50% totally agreed that they think other farmers should use SMS to access/use farm input information, and 34.17% totally agreed that using SMS made their agricultural activities seem more relevant.

#### Simplicity

The third hypothesis H3 (Simplicity has a positive impact on the use of SMS-based farm input information) was removed from all models. This finding was not consistent with the literature for which this construct was a potential driver for adoption of farm input information [2], perceived ease of use [99], and associated with use of mobile data services in China [97]. The present results can be explained by the fact that Tunisian small-scale farmers’ responses to the questions related to simplicity items were not congruent. More than half of the farmers (59.62%) from the total sample did not agree at all that they had no difficulty finding the information they wanted when using SMS. However, almost all farmers for the three studied samples (94.77%) agreed that they had no difficulty understanding how to manage the use of SMS. In addition, a large proportion of all farmers agreed (30.40%) or totally agreed (48.69%) that they had no difficulty implementing the information they obtained when using SMS.

#### Observability

The fourth H4 (Observability has a positive impact on the use of SMS-based farm input information) was supported for the livestock and beekeeping models. These findings were consistent with the literature review [2] where this construct was confirmed as a driver for adopting agricultural input information and had the strongest effect on use of ICT by respondents. [35] also argues that the interaction between early adopters and others has the strongest effect on farmers’ use of ICT for agricultural input information. [95] mentions observability as a factor affecting the use or adoption of innovative technology. Other authors argue that this factor has a significant effect on the intention of adoption of potential users [53,55,97,101]. In developing countries like Tunisia, interpersonal and informal sources of information were the most preferred, trusted, and used by small-scale farmers [2]. Neighbours, relatives, friends, and other farmers are frequently the main sources of information used by small farmers [29].

#### Social influence

The fifth H5 (Social influence has a positive impact on the use of SMS-based farm input information) was only supported for the livestock model. For the livestock model, the small-scale livestock keepers stated that their neighbours, friends, and relatives used SMS technology which gave them a particular status (sense of prestige). This result is consistent with hat of [51,57,102], who all found a significant positive effect between behavioural intention to use and actual usage/adoption of ICT. The results indicate that if small-scale holders have a strong intention to use mobile applications in their farming activities through the positive effect of the social influence, then they are most likely to use them [4] Farmers contemplate that their economic status will be perceived better by others who are important to them if they adopt SMSs. Due to social influence, farmer users favourably perceive the SMSs as an easy-to-use tool for agriculture. It also makes them believe that their financial situation will improve [103]. However, our result for the livestock model is not consistent with that of [2].

#### Information quality

The sixth hypothesis H6 (Information quality has a positive impact on the use of SMS-based farm input information) was supported for the olive crop and beekeeping models. The empirical findings reveal that most beekeepers and olive farmers declared that the information received from SMSs was relevant and suitable for their current needs, both in format and quantity. This result was consistent with the literature, in which this construct was found to be a key driver in the use of ICT in agricultural input information [63]. In this context [2], argues that the construct plays a determinant role in the use of ICT for agricultural input information by cereal farmers. [104] highlighted that information quality was a determinant of the effectiveness of ICT tools in the dissemination of agricultural information in Pakistan. [105] considered information quality to be a major factor in the use of ICT services in developing countries. [106,107] both found a positive effect of information quality on the use and adoption of ICTs in the agricultural sector.

#### SMS increased adoption

The seventh hypothesis H7 (Use of SMS to increase the adoption of farm input information has a positive impact on adoption of farm input information) was supported for the three considered models. This construct was consistent with previous published results. On one hand, [2] argues that that use of ICT-based farm input information has a positive effect on increased adoption of the information. On the other hand, increased adoption is the result of the use of ICT in agricultural input information [108,109]. In Tunisia, small-scale farmers stated that after they had started using SMS, they found it easier to access and use farm input information; this is consistent with our results given that 74.58% of the sample declared that they kept SMSs as an information reference [29].

## Conclusion and policy implications

The purpose of this study is to identify the factors affecting the adoption of Short Message Service (SMS) through a contextual ICT model for livestock, olive crop, and beekeeping. This research showed that 5 drivers affect the use of SMS by small-scale livestock keepers (compatibility, relative advantage, social influence, observability, and information quality), 3 factors for olive tree farmers (computability, relative advantage, and information quality) and 2 drivers for beekeepers (observability and information quality).

This research indicates that the factors affecting the use of SMS (USMS) and increased adoption of SMS (INCADOP) differed according to the typology of the considered farming systems although the farm input information sent by SMS to farmers was specific to the agricultural activities in terms of content, frequency, and quantity. The most significant factor affecting USMS was observability (farmers’ influence on each other) for small-scale livestock farmers, compatibility (farmer’s perception of SMS) for olive crop smallholders, and information quality for beekeeping (challenges faced by farmers in use of SMS). Nevertheless, some aspects need more investigation, particularly the relationship between the factor affecting the USMS and the farming systems context (e.g. Cereal farmers, olive farmers, and breeders). These proposed models can be applied in other contexts, and the proxy questions to measure the constructs can be further improved as there were some inconsistencies in the farmers’ responses for certain constructs. For future research, it is recommended that data be gathered from a larger sample disaggregated by gender to test moderating variables such as farmers’ education and ICT skills.

The analysis of current ICT-based farm input information in Tunisia, leads to recommendations relevant to government agencies and ICT developers concerned with future use and adoption of the SMS system:

- Almost 46% of farmers had at most the primary level of education. In this sense, Tunisian decision-makers should consider the capacity of farmers to understand and take advantage of agricultural input information through SMS. In this sense, a better understanding of the local conditions and farmers’ ability to internalise advice is required to make better use of SMS as a development tool.
- Almost 70% and 23% of farmers had a network problem and phone storage problem respectively. Taking advantage of the opportunities provided by SMS depends on increased connectivity of farmers in rural areas. This suggests the urgent need to improve the digital access by small-scale farmers with technological advances and skills improvement.
- 84% of farmers were not members of an association/cooperative. The relevance of the information provided through SMS is critical to increase use of this technology given that SMS is also a great opportunity for farmer cooperatives to inform their members (e.g., market opportunities and weather hazard warning).
- The extension services were ranked by farmers as the fifth most important source of agricultural input information. The agriculture extension system should engage specialists through an effective mechanism ensuring that agriculture extension information provided by any entity should be designed such that it is close to the ground reality, useful in the right format, timely, and diffused in clear language. Another way to stimulate adoption of ICT in agriculture could be by establishing pioneer farms (leaders-adopters) that can demonstrate uses of ICT, thereby increasing farmers’ perception of technologies’ usefulness and its ease of use.

## Supporting information

S1 Appendix (DOCX)

S1 File (XLSX)
