# Supplementary material for: A contextual ICT model to explain adoption of mobile applications in developing countries: A case study of Tunisia
Source: PLoS One. 2023 Oct 26;18(10):e0287219. doi: 10.1371/journal.pone.0287219 (PMC10602327; doi:10.1371/journal.pone.0287219)
Supplement: S1 Appendix — (DOCX) [file pone.0287219.s001.docx]

**Appendix A3**

Final models frameworks

**Fig A3.1. Final livestock model.**Source: own elaboration from livestock model results (2022).

**Fig A3.2.Final olive crop model.**Source: own elaboration from olive crop model results (2022).

**Fig A3.3.Final beekeeping model.**Source: own elaboration from beekeeping model results (2022).
